# Immunological Trajectories of White Blood Cells from Adolescence to Adulthood: Description and Determinants

**DOI:** 10.3390/diagnostics11112063

**Published:** 2021-11-08

**Authors:** Isaac Barroso, João Tiago Guimarães, Milton Severo, Vanda Craveiro, Elisabete Ramos

**Affiliations:** 1 Department of Clinical Pathology, São João University Hospital Centre, 4200-319 Porto, Portugal; jtguimar@med.up.pt; 2 EPIUnit, Instituto de Saúde Pública, University of Porto, 4050-091 Porto, Portugal; severo.milton@gmail.com (M.S.); vandacraveiro@gmail.com (V.C.); eliramos@med.up.pt (E.R.); 3 Department of Biomedicine, Faculty of Medicine, University of Porto, 4200-319 Porto, Portugal; 4 Department of Public Health and Forensic Sciences, and Medical Education, Faculty of Medicine, University of Porto, 4200-319 Porto, Portugal

**Keywords:** immune response, white blood cells, cohort study, adolescence, adulthood

## Abstract

Background: The immune system gradually matures early in life in the face of internal and external stimuli. Whether the immune responses are lasting and stable during the course of life is still unclear. Methods: As part of the EPITeen cohort, 1183 adolescents were prospectively evaluated at the ages of 13, 17, 21, 24 and 27. Sociodemographic, behavioral and clinical data were collected by self- and face-to-face-administered questionnaires, along with a physical examination comprising anthropometric measurements and blood sample collections. Mixed-effects models were used to identify individual trajectories of white blood cells (WBC) and finite Gaussian mixture models were used to identify the clusters of individual trajectories. Results: Participants were allocated into six clusters based on the individual trajectories of WBC distribution. *Higher* *Inflammatory Activation Cluster* (11.4%) had the highest total WBC count and neutrophils percentage, as well as the lowest percentage of lymphocytes. These participants had significantly higher odds of being overweight [OR = 2.44, 95%CI:1.51–3.92]. *Lowest Levels of WBC Cluster* (24.1%) had the lowest total WBC count, being characterized by a higher participation on sports [OR = 1.54, 95%CI:1.12–2.13]. *Highest Proportion of Eosinophils Cluster* (20.1%) had the highest eosinophils percentage and the highest likelihood of having been diagnosed with a chronic disease [OR = 2.11, 95%CI:1.43–3.13], namely “asthma or allergies” [OR = 14.0 (1.73, 112.2]. *Lowest Proportion of Eosinophils Cluster* (29.1%) had the lowest percentage of eosinophils and basophils, as well as the highest lymphocyte proportion. Participants in the *Undefined* *Cluster* (13.8%) showed the highest percentage of monocytes and basophils and were also characterized by significant lower odds of having parents with 7–9 years of schooling [OR = 0.56, (0.32, 0.99]. Conclusions: In this study we identified distinct immunological trajectories of WBC from adolescence to adulthood that were associated with social, clinical and behavioral determinants. These results suggest that these immunological trajectories are defined early in life, being dependent on the exposures.

## 1. Introduction

The immune system gradually matures during childhood to adolescence [1,2]. Being a complex sensory and adaptative system it comprises a coordinated interplay of various cell populations, providing a balanced response for internal and external stimuli [3,4,5]. These stimuli trigger the development of immune responses early in life in the maturing immune system [1,2,6]. Whether these immune responses are lasting and stable during the course of life is still unclear. The literature is relatively scarce on how the individual’s immune response is influenced by these external factors, namely whether this response is adaptive to stimulus (producing high levels of intraindividual variation) or if there are distinct immune-response patterns, which are relatively stable over time (low levels of intraindividual variation) [4,7,8,9,10]. Among the external stimuli, social and environmental determinants of health, which include socioeconomic, demographic, environmental determinants, along with the health system, may play an important role in modulating the immune system [4,8,11,12,13,14]. These determinants may influence and shape an individual´s immune-cell population frequencies, defined as the individual´s ‘immunotype’, and predict a diverse set of functional responses [9,15,16,17]. On the other hand, recent evidence suggests that white blood cells (WBC) act in a coordinated fashion and their balance along with their subpopulations influences the function of immune system as a whole [9,18]. Since WBC are reliable markers of the immune response and their relative distribution affects the immune system globally, some epidemiological studies have assessed the relative frequency of WBC subpopulations in healthy individuals, however, studies that assess these immune cells responsiveness over a long time period are scarce [8,18,19,20]. Therefore, herein, we sought to assess whether distinct immunological trajectories of WBC develop in a stable manner over a long time period, from adolescence to adulthood, predicting functional responses. Additionally, we also intended to assess determinants of health that condition the development of the immunological response. For that purpose, we prospectively assessed total and differential WBC in a young, healthy population from adolescence to adulthood.

## 2. Material and Methods

Participants were adolescents from the *Epidemiological Health Investigation of Teenagers* in Porto (EPITeen). As already reported elsewhere [21], the population-based EPITeen cohort recruited adolescents born in 1990, who were enrolled at public and private schools in Porto, Portugal. These participants were evaluated at the ages of 13 (2003–2004), 17 (2007–2008), 21 (2011–2013), 24 (2014–2015) and 27 years (2017–2018).

### 2.1. Data Collection

In the first and second study waves, participants were evaluated at schools, and in the subsequent evaluations, they were invited to come to our University Department. Data collection was based on self-administered and face-to-face-questionnaires answered by the participants and by their parents. A physical examination comprising anthropometric measurements and the collection of a blood sample was performed. All procedures were standardized throughout time and performed by a team of trained health professionals.

### 2.2. Blood Measurements

A venous blood sample was drawn after an 8 h overnight fast. All the samples were analyzed at the central laboratory of the São João University Hospital Centre. Total and differential white blood cells (WBC)—neutrophils, monocytes, lymphocytes, eosinophils and basophils—were obtained using an automated blood counter Sysmex^®^ XE-5000 (Sysmex Corp., Kobe, Japan). Serum *high-sensitivity* C-Reactive Protein (*hs*-CRP) was determined through particle-enhanced immunonephelometry using an autoanalyzer Dade Behring Nephelometer II^®^ (Siemens AG, Munich, Germany).

### 2.3. Covariates

At 13 years old, adolescents’ clinical information was obtained by asking to the parents and on the other waves information was reported by the participant. We evaluated the use of chronic medication in the previous 12 months and the presence of chronic disease was assessed asking if they had a disease requiring regular medical care (yes/no). Additionally, specified questions were asked about previous diagnoses of allergy and/or asthma and/or rhinitis separately for each of those three diseases. At 13 years this information was obtained from the parents. Data from 13- and 17-year-old participants was combined, and participants were classified as having a chronic disease if they reported a chronic disease in at least one of the evaluations. For this study, the variable allergic disease was created, corresponding to the diagnosis of at least one of these conditions: allergy, asthma, or rhinitis. For allergy, asthma, and rhinitis, the missing category included participants who did not know if they had the respective disease.

Parental education was based on the highest number of years they had successfully completed formal education, and participants were classified according to the parent with the highest level of education.

Practice of sports was a yes-or-no question considering the practice of sports in addition to the compulsory school curriculum, regardless of frequency or intensity. Data from the evaluation at 17 years was used when there were missing values for practice of sports at 13 years. Leisure-time physical activity was evaluated according to a closed four-choice question of increasing intensity categories: mainly sitting, mainly standing, active or very active [22].

Weight and height were obtained with the subject in light indoor clothes and no shoes. Weight was measured in kilograms, to the decimal, using a digital scale Tanita TBF-300 (Tanita Corp., Tokyo, Japan), and height was measured in centimeters, to the decimal, using a stadiometer 213I (Seca GmbH, Hamburg, Germany). Body mass index (BMI) at 13 years was calculated as weight (kilograms) divided by squared height (meters) and was standardized according to the sex- and age-specific-growth reference data for ages 5-19 years, from the world health organization (WHO) [23]. Participants were described as underweight if the z-score BMI < −2, normal weight if the z-score BMI ≥ −2 & ≤+1, overweight if the z-score BMI > +1 & ≤+2, and obese if the z-score BMI > +2. For BMI, the missing values at 13 years were maintained.

### 2.4. Subjects

At the first recruitment (2003–2004), 2786 eligible participants were identified and 2159 agreed to participate, resulting in an overall participation rate of 77.5%. In the second wave, 1716 (79.5%) of the baseline participants were re-evaluated at 17 years and 783 new participants were integrated in the cohort since they moved to the schools of Porto. From the entire cohort, 1764, 1094 and 1244 participants were re-evaluated at 21, 24 and at 27 years, respectively. WBC measurements from evaluations with concentrations of *hs*-CRP ≥ 10 mg/L were excluded from the analysis, since it constitutes a marker of acute infection [24]. In the present work, we included participants with valid WBC measurements in at least 3 of the 5 evaluations. Thus, our final sample was composed of 1183 subjects. Included participants had higher proportions of chronic medication, parental education and individuals practicing sports, however, reported being less active during leisure-time; additionally, among those included, the proportion of obese participants was lower than the nonincluded participants (Table 1).

### 2.5. Statistical Analysis

To identify the (individual) trajectories for total and differential WBC, we started by testing if the variables (total WBC and each of the subtypes) had a linear, quadratic or cubic trend with time; we found that, except basophiles which were linear, the others were quadratic. Then, to extract the individual trajectories in time for each variable, several parametrizations of mixed-effects models [25] were tested by including step-by-step random intercept, slope, and quadratic terms. We used finite Gaussian mixture models [26] to identify the clusters of individual trajectories with the same characteristics within clusters, and different characteristics between clusters. We used the random effects extracted in the previous step from the final mixed model as variables in mixture models. Finally, the number of clusters was chosen according to the Bayesian Information Criterion (BIC), assuming the lower the BIC, the better the solution was.

To visualize the expected value for the trajectories of each differential and total WBC, a mixed-effect model with linear and quadratic terms was fitted.

A chi-squared test was used to compare proportions. Associations between the clusters identified and the subjects’ characteristics at 13 years were estimated using odds ratio (OR) and 95% confidence intervals (95% CI) calculated by multinomial logistic regression. The final models were adjusted for sex and parental education.

Statistical significance was considered with a significance level of 0.05. The packages nlme and mclust from the R software v.3.6.0 were used to estimate the mixed-effects models and the finite Gaussian mixture models, respectively [27]. Other statistical analysis was performed using IBM^®^ SPSS^®^ Statistics version 25.0 (IBM Corp., Armonk, NY, USA).

### 2.6. Ethical Considerations

The study complies with the Declaration of Helsinki and was approved by the National Commission of Data Protection and by the Ethics Committee from the São João University Hospital Centre and from the Institute of Public Health of the University of Porto. Procedures to guarantee data confidentiality and protection were assured. Parents and adolescents received written and oral information explaining the purpose and the design of the study, and written informed consent was obtained from both when the participants were minors. For waves when they were at least 18 years of age, the consent was only obtained from the participants.

## 3. Results

Participants were allocated into six clusters based on the individual leukocyte distribution trajectories of the 1183 participants who fulfilled the inclusion criteria. The probability of belonging to the most likely cluster ranged from 0.79 to 0.98, showing a good cluster´ identification.

**Figure 1:**
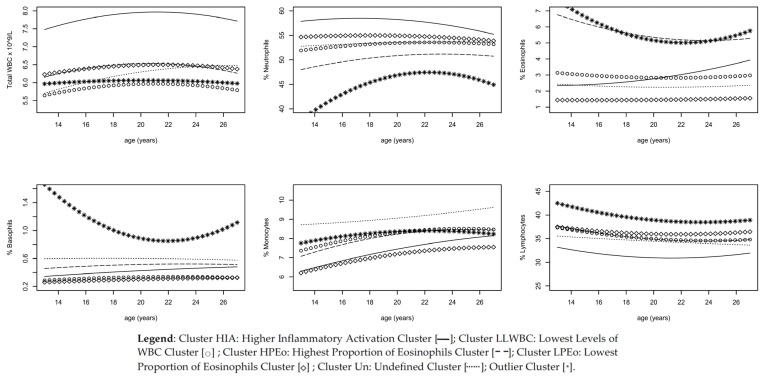
Clusters according to total and differential white blood cells (WBC) trajectories.

One of the clusters was composed of only 17 participants and was not considered for further analyses since they were considered probable outliers due to extreme values in at least one of the evaluations. Thus, our final analysis included 1166 participants. The clusters were named according to a distinctive characteristic, except for one that was named as undefined: *Higher Inflammatory Activation Cluster* (Cluster HIA); *Lowest Levels of WBC Cluster* (Cluster LLWBC); *Highest Proportion of Eosinophils Cluster* (Cluster HPEo); *Lowest Proportion of Eosinophils Cluster* (Cluster LPEo); *Undefined Cluster* (Cluster Un). Figure 1 describes total and differential WBC according to the identified clusters (Table A1 and Table A2). Overall, subjects in *Cluster HIA* (*n* = 136) had the highest total WBC count and percentage of neutrophils, as well as the lowest percentage of lymphocytes, while participants in *Cluster LLWBC* (*n* = 285) had the lowest total WBC count without significant differences regarding the distribution of the differential WBC. The participants in the *Cluster HPEo* (*n* = 238) showed the highest eosinophils proportion and lowest percentage of neutrophils, while those in *Cluster LPEo* (*n* = 344) had the lowest percentage of eosinophils, basophils and monocytes, as well as the highest lymphocyte proportion. Participants in *Cluster Un* (*n* = 163) showed the highest percentage of monocytes and basophils.

Clusters according to sociodemographic and health characteristics are drawn in Table 2. *Cluster HIA* showed a significantly higher percentage of individuals with preobesity and obesity. *Cluster LLWBC* had a significantly higher percentage of individuals practicing sports and higher percentage of participants reporting being very active in leisure-time physical activity. *Cluster HPEo* showed a significantly higher percentage of individuals with chronic disease, allergy, asthma and rhinitis. *Cluster LPEo* had the highest percentage of females and fewer individuals who reported allergic disease. *Cluster Un* had a higher percentage of participants with highest parental education, though without statistical significance.

The results of the crude and adjusted multinomial logistic regression are depicted in Table 3. Female participants had significantly lower odds of being in any cluster. In the crude model, the subjects in *Cluster HIA* had significantly higher odds of being overweight [OR = 2.44, 95%CI:1.51–3.92]. Participants in *Cluster LLWBC* and *Cluster HPEo* showed significantly higher odds of practicing sports, [OR = 1.54, 95%CI:1.12–2.13 and OR = 1.43, 95%CI:1.02–2.00, respectively]. Moreover, participants in *Cluster HPEo* also showed significantly higher odds of having chronic disease [OR = 2.11, 95%CI:1.43–3.13] or allergic disease [OR = 14.0, 95%CI:1.73–112.2]. Participants in *Cluster Un* had significantly lower odds of having parents with 7–9 years of schooling [OR = 0.56, 95%CI:0.32–0.99]. After the adjustment for sex and parental education, participants in *Cluster HIA* had significantly higher odds of being overweight [OR = 2.38, 95%CI:1.47–3.86]. Subjects in *Cluster HPEo* showed significantly higher odds of having chronic disease [OR = 2.08, 95%CI:1.39–3.10], and allergic disease [OR = 3.54, 95%CI:2.45–5.10].

## 4. Discussion

In this study, we were able to identify distinct WBC trajectories from adolescence to adulthood. To the best of our knowledge, this is the first study to prospectively assess these immune cells, identifying different subpopulations of healthy individuals with distinct immunological trajectories of WBC, assessed from 13 to 27 years of age.

The consistency of the relative proportion of each subpopulation suggests that there is an immune-response pattern defined in the first years of life, or even in the prenatal period, that is relatively stable over the years. Since the different leukocyte populations operate in a tightly coordinated fashion with direct interactions with several other immune mediators, their relative distribution influences the immune-system function as a whole [9]. These results corroborate previous studies which have found that despite the considerable interindividual variation in the frequency of leukocytes subsets, the immune profile of each individual is remarkably stable [7,8,28]. However, unlike these studies in which the evaluation period did not exceed several months, this study carried out a longitudinal analysis over a 14-year period, in healthy individuals.

The results regarding the association between the trajectories and the characteristics of the participants, give us a potential explanation for these clusters supporting the causal relationship. The *Higher Inflammatory Activation Cluster* was associated with a higher pattern of immunological activation, though within normal range, with the highest levels of total WBC and percentage of neutrophils as well as the lowest percentage of lymphocytes. Participants included in this cluster also have higher odds of being overweight and obese, even after adjustment for sex and parental education, a proxy of the socioeconomic status. The underlying mechanism linking weight gain with an increase in total WBC levels is not fully understood, however the potential proinflammatory effect of adipose tissue is well known. The high levels of circulating leukocytes in the obese phenotype may reflect the response to stimulation of the inflammatory mediators produced by adipocytes [29,30,31]. In addition, this proinflammatory environment promotes increased levels of neutrophils which, in turn, secrete several proinflammatory mediators, contributing to the increase in systemic inflammation [32,33,34,35]. Leptin may also be involved since it stimulates myeloid differentiation, decreasing after weight loss [36,37]. On the other hand, the lymphocytes represent the regulatory arm of the immune system, are involved in the suppression of inflammation, and their levels are reduced with the increase in systemic inflammation [32,33].

The *Lowest Levels of WBC Cluster* was associated with an immunoprotective pattern of response, with the lowest total WBC levels but without significant differences regarding the proportion of each subpopulation. These results may be explained by the higher physical activity reported by the members of this cluster. Previous studies have shown that high levels of physical activity have been associated with reduced systemic inflammation and reduced total WBC levels in both men and women, regardless of the initial weight [38,39,40,41]. In fact, exercise may lower WBC count through its direct impact on bone marrow hematopoiesis in addition to reducing the trafficking of leukocytes between secondary lymphoid organs and blood [39,42].

The *Highest Proportion of Eosinophils Cluster* was characterized by the highest percentage of eosinophils and a high proportion of basophils, a characteristic allergic response pattern [43], which is in agreement with the clinical characteristics of the participants belonging to this cluster. In fact, in asthma and other allergic diseases the levels of the eosinophils and basophils are observed to be considerably increased [43]. After eosinophils’ activation in the air tract due to pharmacological, hormonal, infectious, or environmental stimuli, a Th2-driven immune response is initiated, which causes airway hyper-responsiveness and chronic remodeling [43,44]. Moreover, eosinophils secrete cytokines, chemokines and cytotoxic proteins that contribute to the activation of macrophages along with the degranulation of mast cells and basophils, triggering the release of various allergic mediators, such as histamine and TNF-α [44,45]. Therefore, the increase in basophils observed in asthma and other allergic diseases also promotes the progression in the allergic reactions, since these cells migrate from the blood compartment to inflamed tissues, acting as inflammatory mediators [45,46]. On the contrary, the *Lowest Proportion of Eosinophils Cluster* was characterized by the lowest percentage of eosinophils and basophils, having the lowest proportion of allergic disease. In this sense, individuals belonging to this cluster seem to have a contrasting immune-response pattern compared with those in *Cluster HPEo*.

Those who belong to the *Undefined Cluster* were characterized by the highest percentage of monocytes and basophils and a considerable increase in the total WBC levels. Since this cluster does not have distinctive characteristics, it probably aggregates all those that do not have a defined pattern.

Taken together, these results show that immunological trajectories of WBC are already developed in adolescence and are stable into adulthood. Furthermore, these results support recent evidence which suggests that extrinsic factors are major drivers of inter-individual variation in the immune system, despite the extent of these differences between healthy individuals being largely unknown [3,4,7,47]. This immune-system diversity generates multiple immunotypes, which are remarkable stable over time in individuals when compared to interindividual heterogeneity [3,4]. In this conception, the individual immune phenotype would constitute a “stability island” in which the immune parameters remain relatively stable. [3,7,28]. Therefore, it is conceivable that the exposures to which one is subjected would be crucial for the individual´s immune-system development, promoting distinct immunological trajectories. Here, we add new insights upon the development and stability of the immunological trajectories of WBC in the long term.

This study should be interpreted in light of some limitations. We lost some participants over time, as some refused to give a blood sample. Included participants tend to have a healthier profile than excluded participants (presented a higher proportion of chronic medication, higher parental education, higher proportion of individuals practicing sports and lower proportion obese people). Although this selection bias may prevent knowledge of the prevalence of each cluster, it does not change its characteristics and the association with sociodemographic and clinical characteristics. Additionally, the bias may have an impact on hiding other potential clusters, since it contributes to creating a more homogenous sample than the population. Furthermore, the absence of leukocyte immunophenotyping does not allow the evaluation of specific WBC subsets, and therefore, hinders a deeper characterization of the studied population. On the other hand, the relatively large and apparently healthy sample is one of the strengths of this study, since most studies are conducted in small and nonhealthy samples. In addition, the 14 years of follow-up makes this study a unique opportunity to longitudinally understand a key period of life—the transition from adolescence to adulthood. In fact, the immune system gradually matures during infancy until adolescence, this life period being decisive in immune-system development, shaping the response patterns observed in adulthood [10,48]. Moreover, since at the age of 27 it is usually too early for the onset of chronic diseases, most participants had not undergone pharmacological therapy, which could have compromised the findings of the study. Therefore, our results allow us to infer causality and may be fairly helpful for stating future research hypotheses.

## 5. Conclusions

In this study were identified distinct, lasting and stable immunological trajectories of WBC from adolescence to adulthood. These results do suggest that these immunological trajectories are defined early in life and may determine and predict the functional responses observed in adulthood. Moreover, our results show that these immunological trajectories are conditioned by the stimuli in which the individual is exposed, leading to the modulation of the immune system, in order to adapt it to the external requirements. To the best of our knowledge this is the first study to longitudinally identify these patterns of immunological responses in a healthy population. A better understanding of these immunological trajectories developed in early life and the mechanisms by which external stimuli may influence and shape the immune repertoire should be crucial to help design new strategies that promote long-term immunological health.

## Figures and Tables

**Table 1:** Comparison of baseline characteristics between included and nonincluded participants.

	Nonincluded		Included		*p*-Value
Characteristics	*n*	%	*n*	%	
	1776	60.4	1166	39.6	
Sex					0.407
Female	921	51.9	586	50.3	
Chronic disease					0.308
Yes	206	19.0	187	20.9	
Missing	690		271		
Allergic disease					0.591
Yes	341	33.1	295	34.3	
Missing/Did not know	745		306		
Allergy					0.496
Yes	265	25.7	234	27.2	
Missing/Did not know	746		306		
Asthma					0.720
Yes	118	11.5	104	12.1	
Missing/Did not know	749		303		
Rhinitis					0.313
Yes	94	9.3	92	10.8	
Missing/Did not know	768		315		
Chronic medication					0.001
Yes	522	51.8	516	59.4	
Missing	768		297		
Parental education					<0.001
≤6 years	553	31.3	221	19.0	
7–9 years	364	20.5	199	17.1	
10–12 years	399	22.5	332	28.5	
>12 years	350	21.0	411	35.3	
Missing	110		3		
Practice of sports					<0.001
Yes	440	44.9	481	55.7	
Missing	797		302		
Leisure-time physical activity					<0.001
Mainly sitting	242	25.4	267	32.5	
Mainly standing	227	23.8	168	20.5	
Active	255	26.8	275	33.5	
Very active	229	24.0	111	13.5	
Missing	823		345		
Body mass index					0.025
Underweight	19	1.7	9	1.0	
Normal weight	783	69.3	641	70.7	
Preobesity	212	18.8	193	21.3	
Obesity	116	10.3	64	7.1	
Missing	646		259		

**Table 2:** Comparison of demographic and behavioral characteristics by clusters.

	Cluster HIA	Cluster LLWBC	Cluster HPEo	Cluster LPEo	Cluster Un	*p*-Value
	N (%)	N (%)	N (%)	N (%)	N (%)	
Characteristics	136 (11.7)	285 (24.4)	238 (20.4)	344 (29.5)	163 (14.0)	
Sex						<0.001
Female	68 (50.0)	104 (36.5) *	94 (39.5) *	231 (67.2) *	89 (54.6)	
Male	68 (50.0)	181 (63.5) *	144 (60.5) *	113 (32.8) *	74 (45.4)	
Chronic disease						<0.001
No	110 (82.1)	230 (36.5)	164 (68.9) *	281 (82.4)	135 (83.3)	
Yes	24 (17.9)	49 (63.5)	74 (31.0) *	60 (17.5)	27 (16.7)	
Allergic disease						<0.001
No	94 (70.1)	190 (69.1)	113 (47.7) *	255 (76.1) *	110 (69.2)	
Yes	40 (29.9)	85 (30.9)	124 (52.3) *	80 (23.9) *	49 (30.8)	
Allergy						<0.001
No	100 (74.1)	206 (74.9)	136 (57.4) *	276 (82.1) *	124 (78.0)	
Yes	35 (25.9)	69 (25.1)	101 (42.6) *	60 (17.9) *	35 (22.0)	
Asthma						<0.001
No	121 (91.0)	249 (89.6)	170 (72.6) *	312 (93.4) *	146 (91.8)	
Yes	12 (9.0)	29 (10.4)	64 (27.4) *	22 (6.6) *	13 (8.2)	
Rhinitis						<0.001
No	125 (92.6)	248 (89.5)	197 (83.8) *	317 (93.8)	151 (95.5)	
Yes	10 (7.4)	29 (10.5)	38 (16.2) *	21 (6.2)	8 (5.0)	
Chronic medication						0.737
No	42 (40.8)	80 (40.2)	71 (36.8)	107 (42.3)	53 (43.8)	
Yes	61 (59.2)	119 (59.8)	122 (63.2)	146 (57.7)	68 (56.2)	
Parental education						0.201
≤6 years	33 (24.3)	45 (15.8)	37 (15.5)	69 (20.1)	37 (22.8)	
7–9 years	20 (14.7)	45 (15.8)	40 (16.8)	71 (20.7)	23 (14.2)	
10–12 years	37 (27.2)	92 (32.4)	71 (29.8)	93 (27.1)	39 (24.1)	
≥12 years	46 (33.8)	102 (35.9)	90 (37.8)	110 (32.1)	63 (38.9)	
Practice of sports						0.044
No	61 (45.5)	104 (37.0)	92 (38.9)	161 (47.5)	75 (46.6)	
Yes	73 (54.5)	177 (63.0)	145 (61.2)	178 (52.5)	86 (53.4)	
Leisure-time physical activity						0.098
Mainly sitting	39 (29.5)	72 (26.3)	54 (23.1)	112 (33.3)	50 (31.8)	
Mainly standing	24 (18.2)	64 (23.4)	51 (21.8)	73 (21.7)	36 (22.9)	
Active	53 (40.2)	84 (30.7)	93 (39.7)	107 (31.8)	47 (29.9)	
Very active	16 (12.1)	54 (19.7)	36 (15.4)	44 (13.1)	24 (15.3)	
Body mass index						0.007
Underweight	0 (0.0)	3 (1.4)	5 (2.5)	1 (0.4)	0 (0.0)	
Normal weight	62 (56.9) *	145 (69.7)	142 (70.6)	195 (75.9)	97 (73.5)	
Preobesity	33 (30.3)	50 (24.0)	39 (19.4)	43 (16.7)	28 (21.2)	
Obesity	14 (12.8)	10 (4.8)	15 (7.5)	18 (7.0)	7 (5.3)	
hs-CRP (median)						0.503
<0.30 mg/L	39 (42.9)	78 (47.3)	76 (43.7)	107 (51.2)	55 (50.5)	
≥0.30 mg/L	52 (57.1)	87 (52.7)	98 (56.3)	102 (48.8)	54 (49.5)	
hs-CRP (mg/L)	Median	Median	Median	Median	Median	0.545
	(25th–75th)	(25th–75th)	(25th–75th)	(25th–75th)	(25th–75th)	
	0.30 (0.20–1.10)	0.30 (0.00–0.70)	0.30 (0.20–0.60)	0.20 (0.10–0.80)	0.20 (0.00–0.70)	

Legend: hs-CRP: high-sensitivity C-reactive protein. hs-CRP median = 0.30 mg/L; Cluster HIA: Higher Inflammatory Activation Cluster; Cluster LLWBC: Lowest Levels of WBC Cluster; Cluster HPEo: Highest Proportion of Eosinophils Cluster; Cluster LPEo: Lowest Proportion of Eosinophils Cluster; Cluster Un: Undefined Cluster; *p*-values < 0.05 are in bold. * post hoc analysis based on residuals of Pearson’s chi-squared test with Bonferroni correction (*p* < 0.05).

**Table 3:** Evaluation of social, clinical and behavioral determinants of the clusters.

	Crude OR (95% CI)				Adjusted * OR (95% CI)			
Characteristics	Cluster HIA	Cluster LLWBC	Cluster HPEo	Cluster Un	Cluster HIA	Cluster LLWBC	Cluster HPEo	Cluster Un
Sex								
Female	**0.49 (0.22, 0.73)**	**0.28 (0.20, 0.39)**	**0.32 (0.23, 0.45)**	**0.59 (0.40, 0.86)**	**0.49 (0.32, 0.73)**	**0.28 (0.20, 0.40)**	**0.32 (0.23, 0.46)**	**0.59 (0.40, 0.87)**
Male	1.00	1.00	1.00	1.00	1.00	1.00	1.00	1.00
Parental education								
≤6 years	1.15 (0.67, 1.98)	0.70 (0.44, 1.12)	0.66 (0.41, 1.08)	0.93 (0.53, 1.54)	1.21 (0.70, 2.08)	0.76 (0.47, 1.22)	0.71 (0.43, 1.17)	0.96 (0.58, 1.59)
7–9 years	0.68 (0.37, 1.24)	0.68 (0.43, 1.08)	0.70 (0.43, 1.12)	**0.56 (0.32, 0.99)**	0.69 (0.38, 1.27)	0.71 (0.44, 1.14)	0.72 (0.44, 1.17)	0.57 (0.33, 1.00)
10–12 years	0.96 (0.58, 1.60)	1.07 (0.72, 1.58)	0.94 (0.62, 1.43)	0.73 (0.45, 1.18)	0.97 (0.58, 1.63)	1.09 (0.73, 1.63)	0.96 (0.63, 1.46)	0.73 (0.45, 1.19)
≥12 years	1.00	1.00	1.00	1.00	1.00	1.00	1.00	1.00
Chronic disease								
No	1.00	1.00	1.00	1.00	1.00	1.00	1.00	1.00
Yes	1.02 (0.61, 1.72)	1.00 (0.66, 1.51)	**2.11 (1.43, 3.13)**	0.94 (0.57, 1.54)	1.03 (0.61, 1.75)	0.97 (0.64, 1.49)	**2.08 (1.39, 3.10)**	0.95 (0.58, 1.57)
Allergic disease								
No	1.00	1.00	1.00	1.00	1.00	1.00	1.00	1.00
Yes	2.25 (0.46, 10.9)	0.96 (0.37, 2.48)	**14.0 (1.73, 112.2)**	1.38 (0.40, 4.72)	1.37 (0.87, 2.15)	1.44 (0.99, 2.08)	**3.54 (2.45, 5.10)**	1.45 (0.95, 2.21)
BMI								
Underweight and normal weight	1.00	1.00	1.00	1.00	1.00	1.00	1.00	1.00
Preobesity and obesity	**2.44 (1.51, 3.92)**	1.30 (0.86, 1.97)	1.18 (0.77, 1.80)	1.16 (0.72, 1.88)	**2.38 (1.47, 3.86)**	1.21 (0.79, 1.86)	1.10 (0.71, 1.71)	1.15 (0.71, 1.87)
Sports practice								
No	1.00	1.00	1.00	1.00	1.00	1.00	1.00	1.00
Yes	1.08 (0.73, 1.62)	**1.54 (1.12, 2.13)**	**1.43 (1.02, 2.00)**	1.04 (0.71, 1.51)	0.97 (0.64, 1.48)	1.20 (0.85, 1.69)	1.12 (0.79, 1.61)	0.92 (0.62, 1.36)

Legend: BMI: body mass index; CI: confidence interval; OR: odds ratio; Bold values represent statistically significant results; Cluster HIA: Higher Inflammatory Activation Cluster; Cluster LLWBC: Lowest Levels of WBC Cluster; Cluster HPEo: Highest Proportion of Eosinophils Cluster; Cluster Un: Undefined Cluster; * adjusted for sex and parental education.

## Data Availability

Data is contained within the article.

## Appendix A

**Table A1:** Description of the total and differential white blood cells (WBC) at 13, 17, 21, 24 and 27 years of age according to the clusters.

	Total *n* = 1183	Cluster *HIA* *n* = 163	Cluster *LLWBC* *n* = 136	Cluster *HPEo* *n* = 285	Cluster *LPEo* *n* = 344	Cluster *Un* *n* = 238
**Total WBC ^a^**	Mean (SD)	Mean (SD)	Mean (SD)	Mean (SD)	Mean (SD)	Mean (SD)
13	6.15 (1.50)	7.49 (2.09)	5.61 (1.04)	6.15 (1.32)	6.16 (1.36)	5.83 (1.37)
17	6.35 (1.51)	7.58 (1.98)	5.91 (1.15)	6.30 (1.41)	6.35 (1.39)	6.09 (1.47)
21	6.56 (1.71)	8.08 (2.00)	5.98 (1.17)	6.60 (1.60)	6.56 (1.74)	6.33 (1.65)
24	6.45 (1.71)	8.04 (2.05)	5.86 (1.14)	6.43 (1.58)	6.36 (1.64)	6.54 (1.84)
27	6.35 (1.56)	7.55 (1.88)	5.79 (0.94)	6.25 (1.28)	6.44 (1.69)	6.43 (1.80)
**Trend**	Β (*p*-value)	Β (*p*-value)	Β (*p*-value)	Β (*p*-value)	Β (*p*-value)	Β (*p*-value)
**Age**	0.22 (<0.001)	0.31 (0.043)	0.21 (<0.001)	0.28 (<0.001)	0.17 (0.018)	0.22 (0.032)
**Age ^2^**	−0.005 (<0.001)	−0.007 (0.055)	−0.005 (<0.001)	−0.007 (<0.001)	−0.003 (0.025)	−0.004 (0.105)
**Neutrophils ^b^**	Mean (SD)	Mean (SD)	Mean (SD)	Mean (SD)	Mean (SD)	Mean (SD)
13	52.3 (10.5)	57.9 (9.00)	51.8 (9.00)	47.9 (8.62)	54.5 (9.97)	53.4 (10.8)
17	53.8 (9.06)	58.9 (9.29)	53.9 (8.49)	50.2 (8.41)	55.2 (8.92)	52.9 (8.58)
21	53.7 (8.80)	58.5 (7.36)	53.0 (7.33)	50.7 (8.62)	54.7 (9.29)	53.3 (9.29)
24	53.7 (8.78)	56.8 (7.13)	53.7 (7.63)	51.3 (8.50)	54.7 (9.16)	54.2 (9.24)
27	52.9 (8.16)	55.4 (7.13)	53.0 (6.44)	50.8 (7.24)	53.8 (9.33)	53.2 (8.85)
**Trend**	Β (*p*-value)	Β (*p*-value)	Β (*p*-value)	Β (*p*-value)	Β (*p*-value)	Β (*p*-value)
**Age**	0.95 (<0.001)	1.19 (0.062)	0.82 (0.494)	1.41 (0.002)	0.48 (0.257)	0.33 (0.580)
**Age ^2^**	−0.023 (<0.001)	−0.034 (0.031)	−0.18 (0.076)	−0.030 (0.007)	−0.014 (0.200)	−0.007 (0.645)
**Eosinophils ^b^**	Mean (SD)	Mean (SD)	Mean (SD)	Mean (SD)	Mean (SD)	Mean (SD)
13	3.43 (2.92)	2.47 (1.33)	3.19 (1.52)	6.83 (3.28)	1.45 (0.66)	2.41 (1.27)
17	2.81 (2.13)	2.30 (1.22)	2.77 (1.39)	5.45 (2.34)	1.44 (0.74)	2.28 (1.13)
21	2.86 (1.96)	2.88 (1.34)	2.84 (1.29)	5.36 (2.23)	1.51 (0.75)	2.27 (1.06)
24	3.07 (2.22)	3.58 (1.96)	3.00 (1.38)	5.33 (2.64)	1.55 (0.77)	2.20 (1.07)
27	3.06 (2.18)	3.77 (1.73)	2.89 (1.44)	5.17 (2.45)	1.48 (0.75)	2.39 (1.23)
**Trend**	Β (*p*-value)	Β (*p*-value)	Β (*p*-value)	Β (*p*-value)	Β (*p*-value)	Β (*p*-value)
**Age**	−0.28 (<0.001)	−0.19 (0.142)	−0.19 (0.019)	−0.65 (<0.001)	−0.029 (0.419)	−0.14 (0.076)
**Age ^2^**	0.007 (<0.001)	0.008 (0.019)	0.005 (0.025)	0.014 (<0.001)	0.001 (0.291)	0.003 (0.085)

**Legend**: Cluster HIA: Higher Inflammatory Activation Cluster; Cluster LLWBC: Lowest Levels of WBC Cluster; Cluster HPEo: Highest Proportion of Eosinophils Cluster; Cluster LPEo: Lowest Proportion of Eosinophils Cluster; Cluster Un: Undefined Cluster. ^a^ Absolute levels expressed as Total WBC × 10^9^/L. ^b^ differential count expressed as relative percentage (%).

**Table A2:** Description of the total and differential white blood cells (WBC) at 13, 17, 21, 24 and 27 years of age according to the clusters.

	Total *n* = 1183	Cluster *HIA* *n* = 163	Cluster *LLWBC* *n* = 136	Cluster *HPEo* *n* = 285	Cluster *LPEo* *n* = 344	Cluster *Un* *n* = 238
**Basophils ^b^**	Mean (SD)	Mean (SD)	Mean (SD)	Mean (SD)	Mean (SD)	Mean (SD)
13	0.40 (0.42)	0.36 (0.20)	0.29 (0.16)	0.46 (0.27)	0.25 (0.13)	0.58 (0.38)
17	0.40 (0.24)	0.39 (0.18)	0.32 (0.14)	0.51 (0.25)	0.30 (0.15)	0.61 (0.32)
21	0.41 (0.25)	0.43 (0.21)	0.33 (0.16)	0.49 (0.24)	0.30 (0.16)	0.60 (0.30)
24	0.44 (0.27)	0.48 (0.21)	0.35 (0.15)	0.55 (0.31)	0.32 (0.19)	0.58 (0.25)
27	0.42 (0.26)	0.45 (0.24)	0.32 (0.14)	0.50 (0.27)	0.32 (0.16)	0.59 (0.31)
**Trend**	Β (*p*-value)	Β (*p*-value)	Β (*p*-value)	Β (*p*-value)	Β (*p*-value)	Β (*p*-value)
**Age**	0.008 (0.205)	0.021 (0.155)	0.025 (0.002)	0.029 (0.034)	0.013 (0.009)	0.010 (0.656)
**Age ^2^**	<0.001 (0.386)	<0.001 (0.443)	<0.001 (0.005)	<0.001 (0.066)	<0.001 (0.263)	<0.001 (0.606)
**Monocytes ^b^**	Mean (SD)	Mean (SD)	Mean (SD)	Mean (SD)	Mean (SD)	Mean (SD)
13	7.22 (1.96)	6.52 (1.11)	7.50 (1.63)	7.19 (2.21)	6.41 (1.29)	8.86 (2.29)
17	7.21 (1.79)	6.56 (1.27)	7.65 (1.72)	7.58 (2.01)	6.50 (1.36)	8.15 (1.98)
21	8.31 (2.00)	7.82 (1.60)	8.57 (1.88)	8.57 (1.99)	7.52 (1.63)	9.50 (2.37)
24	8.23 (2.08)	7.85 (1.49)	8.56 (2.17)	8.36 (1.92)	7.40 (1.59)	9.44 (2.56)
27	8.25 (2.03)	8.02 (1.51)	8.41 (1.88)	8.36 (2.00)	7.53 (1.63)	9.41 (2.64)
**Trend**	Β (*p*-value)	Β (*p*-value)	Β (*p*-value)	Β (*p*-value)	Β (*p*-value)	Β (*p*-value)
**Age**	0.34 (<0.001)	0.32 (0.002)	0.41 (<0.001)	0.48 (<0.001)	0.35 (<0.001)	−0.023 (0.876)
**Age ^2^**	−0.006 (<0.001)	−0.005 (0.063)	−0.008 (<0.001)	−0.010 (<0.001)	−0.006 (<0.001)	0.002 (0.545)
**Lymphocytes ^b^**	Mean (SD)	Mean (SD)	Mean (SD)	Mean (SD)	Mean (SD)	Mean (SD)
13	36.5 (9.49)	32.7 (9.31)	37.2 (8.57)	37.6 (8.09)	37.4 (9.41)	34.8 (11.0)
17	35.8 (8.15)	32.7 (8.47)	35.4 (7.82)	36.3 (7.89)	36.5 (8.34)	36.1 (7.89)
21	34.6 (7.93)	30.1 (6.42)	35.1 (6.88)	34.7 (7.97)	35.8 (8.38)	34.1 (8.43)
24	34.4 (8.03)	31.0 (6.24)	34.2 (7.18)	34.3 (7.95)	35.9 (8.46)	33.4 (8.65)
27	35.1 (7.54)	32.1 (6.60)	35.1 (6.29)	35.0 (6.64)	36.6 (8.50)	34.2 (8.36)
**Trend**	Β (*p*-value)	Β (*p*-value)	Β (*p*-value)	Β (*p*-value)	Β (*p*-value)	Β (*p*-value)
**Age**	−1.03 (<0.001)	−1.42 (0.014)	−1.11 (0.003)	−1.31 (0.001)	−0.87 (0.027)	−0.26 (0.650)
**Age ^2^**	0.022 (<0.001)	0.033 (0.210)	0.020 (0.040)	0.027 (0.007)	0.020 (0.040)	0.003 (0.826)

**Legend**: Cluster HIA: Higher Inflammatory Activation Cluster; Cluster LLWBC: Lowest Levels of WBC Cluster; Cluster HPEo: Highest Proportion of Eosinophils Cluster; Cluster LPEo: Lowest Proportion of Eosinophils Cluster; Cluster Un: Undefined Cluster. ^b^ differential count expressed as relative percentage (%).
